# Simulation-Based Medical Education Improves Procedural Confidence in Core Invasive Procedures for Military Internal Medicine Residents

**DOI:** 10.7759/cureus.11998

**Published:** 2020-12-09

**Authors:** Lauren A Sattler, Chad Schuety, Mark Nau, Daniel V Foster, John Hunninghake, Tyson Sjulin, Joshua Boster

**Affiliations:** 1 Internal Medicine, Brooke Army Medical Center, Fort Sam Houston, USA; 2 Pulmonary and Critical Care Medicine, Walter Reed National Military Medical Center, Bethesda, USA; 3 Pulmonary and Critical Care Medicine, Brooke Army Medical Center, Fort Sam Houston, USA

**Keywords:** simulation education, military medicine, procedural competency

## Abstract

Introduction

The American Board of Internal Medicine (ABIM) requires that trainees receive procedural training for certification; however, Internal Medicine (IM) residents perform a variable number of procedures throughout residency training. This results in differences in confidence levels as well as procedural competence. For active-duty military trainees, this is especially problematic, as these procedural skills are often required during deployment soon after residency graduation. This deficit can be improved through standardized simulation-based training.

Methods

All internal medicine residents at our institution were invited to participate in a standardized simulation-based training program for core internal medicine procedures (lumbar puncture, arterial line, central line, thoracentesis, paracentesis, and arthrocentesis). Residents were asked to qualitatively rate their perceived procedural confidence using a Likert scale ranging from 1 (not at all confident) to 5 (extremely confident) in their ability to independently perform core internal medicine procedures prior to the simulation exercise. Experienced senior residents and internal medicine faculty instructed and supervised each resident as they performed the procedures. Following the simulation exercise, the residents repeated the survey and were asked to report whether or not they found the exercise useful.

Results

Of the 96 residents invited to participate, 49 completed the pre-simulation questionnaire and 36 completed the post-simulation questionnaire. The cumulative mean Likert scale confidence rating for all procedures showed a statistically significant improvement post-simulation as compared to pre-simulation, including lumbar puncture (2.45±1.1 vs. 3.42±0.87, p<0.05), arterial line (2.48±1.06 vs. 3.39±1.04, p < 0.05), central line (2.86±1.08 vs. 3.5±1.02, p < 0.05), thoracentesis (2.67±1.10 vs. 3.64±0.83, p < 0.05), paracentesis (3.1±1.08 vs. 3.82±0.74, p < 0.05), and arthrocentesis (2.56±1.07 vs. 3.67±0.80, p < 0.05). All (36/36) trainees reported that they perceived the simulation exercise as valuable.

Conclusion

Internal medicine residents across all post-graduate year (PGY) levels at our institution lacked confidence to independently perform core internal medicine procedures. Utilizing simulation-based medical education as an adjunct to clinical training is well accepted by internal medicine trainees, and resulted in significantly improved procedural confidence. This intervention was well received by trainees and could feasibly be replicated at other active-duty military internal medicine residency programs to assist with readiness. Research is currently in progress to correlate in-situ competency and evaluate clinical outcomes of this improved confidence.

## Introduction

The American Board of Internal Medicine (ABIM) requires that Internal Medicine (IM) residents receive procedural training; however, residents are not mandated to perform all procedures, and only general competence is required [1]. This has led to significant heterogeneity in the procedural skills training that IM residents receive with a consistent emphasis on experiential learning, particularly the “see one, do one, teach one” model [2-4]. However, reported clinical procedural experience has been demonstrated to be an unreliable proxy for procedural skill [5]. Additionally, due to this variability in clinical experience and in the training received during residency, levels of confidence and competence in performing common bedside procedures differ greatly among trainees [6].

This lack of standardized training experience directly impacts patient outcomes due to the invasive nature of these procedures and the associated risk for complications [7-9]. Additionally, for active-duty military IM residents, the lack of procedural confidence is of particular concern because IM specialists and subspecialists often deploy as intensivists with the expectation to maintain procedural competence for deployment readiness per the Air Force’s Comprehensive Medical Readiness Program (CMRP) as well as the Army’s Individual Critical Task List (ICTL).

In order to address these deficits, high-fidelity simulation-based mastery learning (SBML) has been identified as a way to standardize procedural experience and produce measurable quality outcomes [10,11]. SBML has been utilized to improve outcomes in multiple facets of medical training, including communication skills and ultrasound techniques [12,13]. Furthermore, SBML has been used to improve training in advanced cardiac life support (ACLS) due to perceived trainee discomfort and reported deficits [14,15]. Given the success with high-fidelity SBML in these areas of medical training, this teaching technique has been incorporated into procedural training to address these deficits. Significant improvements have been demonstrated in procedural competence and confidence among trainees by using SBML to learn multiple procedures, including central line placement, lumbar puncture, paracentesis, thoracentesis, and arthrocentesis in the civilian sector [16-20]. Given the requirement for procedural competence placed upon active-duty internists, and the emphasis on the use of simulation to augment clinical skills, we explored the utility of simulation in improving perceived procedural confidence in active-duty IM residents.

Due to the demonstrated benefits of high-fidelity simulation and deliberate practice in teaching procedural skill, efforts have been made to use SBML to instruct trainees in core internal medicine procedures [21,22]. However, there remains no standardized, simulation-based curriculum utilized across Internal Medicine residency training programs within the military healthcare system. We report the results of the adoption of a simulation-based medical education curriculum for core IM invasive bedside procedures and its implementation at a single IM residency program for active-duty military resident physicians.

## Materials and methods

All IM residents in post-graduate year (PGY) 1-3 at Brooke Army Medical Center in San Antonio, Texas, were invited to participate in the simulated training course. The course included multiple two-hour sessions that were conducted over two separate days with each session including three of six procedural skills: lumbar puncture, arterial line, central line, thoracentesis, paracentesis and arthrocentesis. Participants were divided into small groups comprised of two or three residents that rotated through each skill station. Experienced senior residents and IM faculty instructed and supervised each resident as they performed the procedures. Skills education and training were performed on a combination of high-fidelity SimMan3G mannequins (Laerdal), CentraLineMan system (Simulab), and Ultrasound ArteriaLine Trainer (Simulab).

Prior to the simulation sessions, the participants completed a questionnaire with non-identifiable demographic information, consent, and perceived procedural confidence level using a Likert scale with 1 correlating with (not at all confident), 2 (not so confident), 3 (somewhat confident), 4 (very confident), 5 (extremely confident) in their ability to independently perform core internal medicine procedures. Following the simulation session, the participants repeated the same questionnaire with an additional question about the overall perceived utility of the skills course. Data from the pre- and post-test survey were compared using Kruskal-Wallis tests with statistical significance set at p < 0.05.

## Results

**Figure 1:**
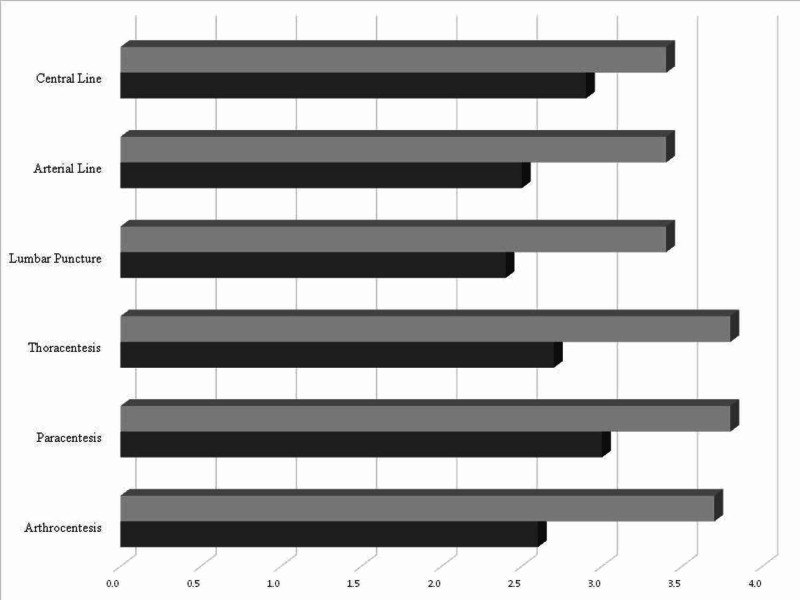
Mean self-reported confidence among residents for each individual core procedure Darker color represents the mean confidence rating prior to the simulation, with the lighter color representing after the simulation

Of the 96 residents invited to participate, 49 completed the pre-simulation questionnaire and 36 completed the post-simulation questionnaire. The group was comprised of 26%, 33% and 41% of residents from the PGY1, PGY2 and PGY3 classes, respectively. All procedures showed a statistically significant improvement post-simulation as compared to pre-simulation, including lumbar puncture (2.45±1.1 vs. 3.42±0.87, p < 0.05), arterial line (2.48±1.06 vs. 3.39±1.04, p < 0.05), central line (2.86±1.08 vs. 3.5±1.02, p < 0.05), thoracentesis (2.67±1.10 vs. 3.64±0.83, p < 0.05), paracentesis (3.1±1.08 vs. 3.82±0.74, p < 0.05), and arthrocentesis (2.56±1.07 vs. 3.67±0.80, p < 0.05). All (36/36) trainees reported that they perceived the simulation exercise as valuable on the post-simulation questionnaire (Table 1; Figure 1). 

**Table 1:** Comparison of self-reported confidence by Likert scale among residents, by PGY year for each individual core invasive procedure PGY, post-graduate year; SD, standard deviation

Pre-Simulation								Post-Simulation						
		1	2	3	4	5	Average±SD	1	2	3	4	5	Average±SD	p-value
Central Line		5	14	16	11	3	2.86 ± 1.08	1	5	15	9	6	3.5 ± 1.02	0.030
PGY1		5	5	1	2	0	1.62 ± 1.08	1	5	2	3	1	3.17 ± 1.19	
PGY2		0	4	10	1	1	2.94 ± 0.19	0	0	7	2	1	3.4 ± 0.7	
PGY3		0	5	5	8	2	3.35 ± 0.99	0	0	6	4	4	3.86 ± 0.86	
Arterial Line		9	17	15	6	2	2.48 ± 1.06	1	5	16	7	7	3.39 ± 1.04	<0.001
PGY1		5	7	1	0	0	1.69 ± 0.63	1	3	4	2	2	2.64 ± 1.24	
PGY2		2	7	5	1	1	2.5 ± 1.03	0	0	7	2	1	3.4 ± 0.69	
PGY3		2	3	9	5	1	3 ± 1.03	0	2	5	3	4	3.64 ± 1.08	
Lumbar Puncture		13	9	21	4	2	2.45 ± 1.1	0	4	18	9	5	3.42 ± 0.87	<0.001
PGY1		6	1	6	0	0	2 ± 1	0	3	3	5	1	3.33 ± 0.98	
PGY2		4	3	8	0	1	2.44 ± 1.09	0	1	6	2	1	3.3 ± 0.82	
PGY3		3	5	7	4	1	2.75 ± 1.12	0	0	9	2	3	3.57 ± 0.85	
Thoracentesis		7	17	17	8	3	2.67 ± 1.1	0	1	20	18	6	3.64 ± 0.83	0.002
PGY1		4	10	5	0	0	2.05 ± 1.17	0	1	9	4	1	3.33 ± 0.91	
PGY2		2	1	9	2	0	2.79 ± 0.91	0	0	5	9	1	3.73 ± 0.74	
PGY3		1	6	3	6	3	3.21 ± 0.9	0	0	6	5	4	3.87 ± 0.85	
Paracentesis		4	12	15	17	4	3.1 ± 1.08	0	1	17	16	11	3.82 ± 0.74	<0.001
PGY1		4	6	5	3	1	2.53 ± 0.71	0	1	7	4	3	3.6 ± 0.72	
PGY2		0	3	5	5	1	3.29 ± 0.89	0	0	5	7	3	3.87 ± 0.59	
PGY3		0	3	5	9	2	3.53 ± 1.23	0	0	5	5	5	4 ± 0.83	
Arthrocentesis		9	15	17	7	2	2.56 ± 1.07	0	2	18	18	7	3.67 ± 0.8	<0.001
PGY1		7	7	4	0	1	2 ± 1.05	0	2	6	4	3	3.53 ± 0.99	
PGY2		2	3	7	2	0	2.64 ± 0.93	0	0	6	9	0	3.6 ± 0.51	
PGY3		0	5	6	5	1	3.18 ± 0.93	0	0	6	5	4	3.87 ± 0.83	

## Discussion

Despite evidence that high-fidelity simulation is an effective, well-received tool for procedural training as well as skills maintenance, a standardized simulation curriculum does not currently exist for training active duty IM residents in core procedures [23,24]. Our study adds to the growing body of evidence that supports the use of high-fidelity simulation and deliberate practice in a structured learning environment to improve trainee procedural confidence. Use of this model has previously been used most commonly to instruct trainees in individual procedures; however, our study demonstrates the feasibility of developing and implementing a curriculum for all core IM procedures. The course has subsequently become a sustainable adjunct to the curriculum at our institution and is held on an annual basis.

An important finding of our study was that significant improvement in procedural confidence was demonstrated consistently for each PGY level, including residents that had completed most of their training. This supports findings of previous studies demonstrating that deliberate practice in a controlled environment is a useful adjunct to procedural learning regardless of experience in the clinical setting [14]. Utilizing simulation-based procedural training as part of a standardized curriculum is of particular importance for military IM residents, as these procedures are often necessary during deployment soon after completion of residency. Consistent implementation of a curriculum such as ours could be useful in mitigating self-identified lack of procedural confidence among trainees [4-6].

Though our simulation curriculum demonstrates a practical model that could be replicated at other training programs, one potential limitation is the resources required. In order to implement this study, we used high-fidelity simulation mannequins from the simulation center at our institution, which may not be available at other institutions. The curriculum also involved supervision by IM faculty, which required approximately two hours of time volunteered for each session. The small-group training model with a high instructor-to-learner ratio was well-received by our residents; however, coordination of these resources may be an obstacle to course implementation at other institutions. This study was additionally limited by the relatively small sample size at a single institution and did not include a validation process for each procedural skill; however, this is a current goal for further study.

These limitations do not lessen the effect of the standardized curriculum on perceived confidence with bedside procedural skills; however, self-assessment does not always directly result in improved clinical performance [25,26]. Further research is needed to determine whether performance of all simulated core IM procedures translates to the clinical environment, including safety outcomes such as procedural complications and associated infections. Prior studies have demonstrated reduced rates of procedure-related complications through deliberate practice in a simulated environment [27-29]. However, these outcomes have primarily been studied in central venous catheter insertion and further outcomes research is needed for many of the other core IM procedures. Lastly, additional studies are needed to determine if deliberate practice during residency improves procedural confidence and competence among active-duty staff IM physicians in the pre-deployment setting.

## Conclusions

Internal medicine residents across all post-graduate year levels at our institution lacked confidence to independently perform core internal medicine procedures. Utilizing simulation-based medical education as an adjunct to clinical training is well accepted by internal medicine trainees, and resulted in significantly improved procedural confidence across all post-graduate levels. This intervention was well received by trainees and could feasibly be replicated at other active-duty military internal medicine residency programs to assist with readiness. Research is currently in progress to correlate in-situ competency and evaluate clinical outcomes of this improved confidence.
